# The C-BIG Repository: an Institution-Level Open Science Platform

**DOI:** 10.1007/s12021-021-09516-9

**Published:** 2021-05-18

**Authors:** Samir Das, Rida Abou-Haidar, Henri Rabalais, Sonia Denise Lai Wing Sun, Zaliqa Rosli, Krishna Chatpar, Marie-Noëlle Boivin, Mahdieh Tabatabaei, Christine Rogers, Melanie Legault, Derek Lo, Clotilde Degroot, Alain Dagher, Stephanie O. M. Dyke, Thomas M. Durcan, Annabel Seyller, Julien Doyon, Viviane Poupon, Edward A. Fon, Angela Genge, Guy A. Rouleau, Jason Karamchandani, Alan C. Evans

**Affiliations:** 1 McGill Centre for Integrative Neuroscience, McGill University, Montréal, Québec, Canada; 2 Montreal Neurological Institute-Hospital, Montreal, Quebec, Canada; 3 McConnell Brain Imaging Centre, Montreal Neurological Institute-Hospital, Montreal, Quebec, Canada; 4 Clinical Research Unit, McGill University, Montreal, Quebec, Canada; 5 Department of Neurology and Neurosurgery, McGill University, Montreal, Quebec, Canada; 6 Early Drug Discovery Unit (EDDU), Montreal Neurological Institute-Hospital, Montreal, Quebec, Canada

**Keywords:** Open Science, Database, Genetic, Registered access, Biobank, Interoperability

## Abstract

In January 2016, the Montreal Neurological Institute-Hospital (The Neuro) declared itself an Open Science organization. This vision extends beyond efforts by individual scientists seeking to release individual datasets, software tools, or building platforms that provide for the free dissemination of such information. It involves multiple stakeholders and an infrastructure that considers governance, ethics, computational resourcing, physical design, workflows, training, education, and intra-institutional reporting structures. The C-BIG repository was built in response as The Neuro’s institutional biospecimen and clinical data repository, and collects biospecimens as well as clinical, imaging, and genetic data from patients with neurological disease and healthy controls. It is aimed at helping scientific investigators, in both academia and industry, advance our understanding of neurological diseases and accelerate the development of treatments. As many neurological diseases are quite rare, they present several challenges to researchers due to their small patient populations. Overcoming these challenges required the aggregation of datasets from various projects and locations. The C-BIG repository achieves this goal and stands as a scalable working model for institutions to collect, track, curate, archive, and disseminate multimodal data from patients. In November 2020, a Registered Access layer was made available to the wider research community at https://cbigr-open.loris.ca, and in May 2021 fully open data will be released to complement the Registered Access data. This article outlines many of the aspects of The Neuro’s transition to Open Science by describing the data to be released, C-BIG’s full capabilities, and the design aspects that were implemented for effective data sharing.

## Introduction

In January 2016, the Montreal Neurological Institute-Hospital (The Neuro) declared itself an Open Science organization (Owens 2016a,b), embodying the principles of Open Science at the institutional level. The Neuro’s plan was to forgo institutional patents, generate institutional support for principal investigators, implement significant procedural changes to existing workflows, and design an infrastructure solution to support these practices across the institute by handling heterogeneous data from multiple research units (Das et al. 2017). Several of these aspects required novel institutional transformation, involving multiple stakeholders and an infrastructure that considers governance, ethics, computational resourcing, physical design, workflows, training and education.

As part of the larger IT ecosystem at The Neuro (Das et al. 2016), the C-BIG repository (C-BIG) was launched internally in 2019 as the Open Science biospecimen and clinical data repository, and publicly in November 2020 at https://cbigr-open.loris.ca to the wider research community. This repository was built using LORIS (Das et al. 2012) to collect biospecimens, and clinical, imaging and genetic data from patients with neurological disease and healthy controls. C-BIG’s architectural design was informed by The Neuro’s Clinical Research Unit (CRU) and their approach towards translational research and clinical care. Creating new tools integrating the CRU’s established and proven workflows with paradigms for reproducible data sharing resulted in an institutional version of LORIS containing a biospecimen system, known as “the Biobank”.

The C-BIG repository was designed to reflect best practices in medical ethics, while allowing for the tracking and dissemination of biospecimen data, raw or derived, and supports metadata and summary statistics at multiple levels of access control, including access for bona fide researchers and clinical care professionals through the novel Registered Access mosdel (Dyke et al., 2016a, b & 2018). C-BIG features current best data sharing practices, such as FAIR (Wilkinson et al. 2016) or COBIDAS (Nichols et al. 2017; Pernet et al. 2020), and includes provenance capture, fully auditable logging, and other standardization efforts (Abrams et al., 2021).

Transitioning from traditional closed access to open practices is still an uncommon endeavour and difficult to advance due to the complexities of effectuating change in established practices, building a supporting infrastructure, and appropriately resourcing the efforts to do so. In many cases, academic incentive structures directly impede such a transition as investigators are rewarded for high-impact publications, instead of being recognized for sharing data (Campbell et al. 2002; Zinner et al. 2016). Competing views on best practices may also hinder consensus. To ensure a successful transition to open sharing, the director of The Neuro engaged in an 18 month stakeholder consultation, involving numerous committees and working groups, formed to address issues including ethics, training, user experience, and infrastructure among others.

The development of C-BIG required significant efforts, and was financially supported by a number of sources including the Neuro itself. A pivotal philanthropic donation from a benefactor interested in advancing open science established the Tanenbaum Open Science Institute (TOSI) (Poupon et al. 2017), while Brain Canada funded supporting grants, such as the Canadian Open Neuroscience Platform (Cavoukian et al. 2019) to assist with data federation technologies. This paper outlines The Neuro’s institutional-level transition to Open Science, by i) describing the data to be released, ii) the various capabilities of C-BIG, and iii) the design and workflow details that were implemented for effective data sharing and scaling.

## Methods

**Fig. 1:**
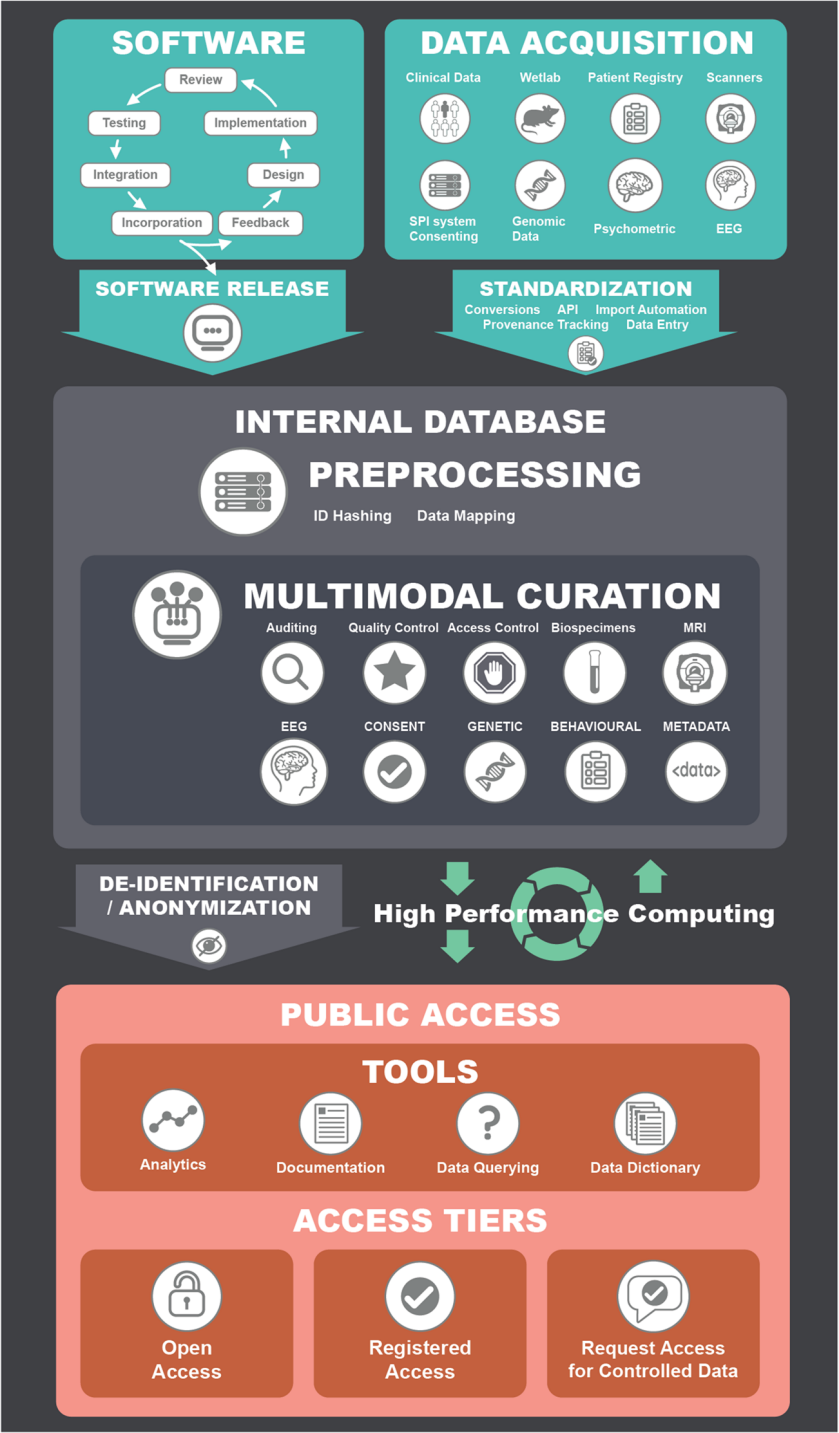
Architectural diagram of the various components used in C-BIG to illustrate the workflows involved in acquiring, curating, processing, and disseminating data: Software: The LORIS platform undergoes continual development with regular releases to improve the functionality, security and interface of the C-BIG repository. Data Acquisition: C-BIG acquires a number of data modalities from consenting patients whose unique IDs are tracked via the SPI patient registry based on patient preference and their risk tolerance. Internal Database: Data are standardized and housed in the C-BIG Internal Database, where metadata/data can be viewed and manipulated by lab technicians and researchers across multiple projects via a web-based repository organizing the metadata in modules specific to their needs. Anonymization: Datasets are then anonymized to reduce the risk of re-identification. High Performance Computing can be leveraged using any HPC system depending on the user preferences. Public Access: Datasets are made available via the public layer where user access is regulated by tiers determined by the nature of the dataset. The data can be queried in a granular manner by researchers wanting to do specific processing and analysis, or seeking summary statistics, documentation or quality control results

The C-BIG repository sits at the centre of TOSI’s goal of openly sharing scientific data. It is a web platform based on complex workflows and technologies cohesively constructed to curate data for broad use by the research community (see Fig. 1 for architecture).

In this work, we first highlight the legacy operations, technologies and workflows at The Neuro prior to its Open Science mission to better demonstrate the full implementation required to allow The Neuro to operationalize C-BIG’s Open Science mandate.

### Legacy Infrastructure

The Neuro’s Clinical Research Unit (CRU) is a high volume, high activity platform, which currently manages more than 100 active clinical trials spanning many disease areas including neurodegeneration, stroke, and brain tumours. This unit was considered a critical platform when The Neuro was designing C-BIG, as it was responsible for the recruitment and consenting process for all new patients. The Neuro’s CRU is somewhat unique in that it is a university endeavor that must organize and harvest data from patient’s clinical records through our healthcare institution (the McGill University Health Centres - MUHC) and provincial electronic medical records to track patient data, imaging analysis, and coordinate multi-disciplinary workflows for numerous and varied clinical populations. In the absence of an institutional database, data was collected in three different locations. The CRU previously relied on Excel spreadsheets to log patient information and their consent status, with records stored on individual computers. They were not synchronized across all instances, typically transferred manually and updated sporadically. After providing consent, a patient’s clinical information was collected using disease-specific paper intake forms, which were then associated with a patient ID. This was followed by the collection of biosamples, that were collected based on the patient’s pathology (and consent). The patient code was handwritten on the containers containing human tissue and fluid samples before being sent to a specimen processing laboratory. The lab staff relied on a proprietary Laboratory Information Management System (LIMS), to store and track the inventory of specimens available in storage facilities. Although a powerful tool, the proprietary software presented several difficulties with regard to its licensing, lack of interoperability, and scaling limitations which hindered The Neuro’s Open Science mission.

Building a comprehensive environment for data sharing and curation requires an open source solution that is extensible and interoperable with cross-modal workflows. The previous infrastructure was built on a piecemeal basis to serve the workflows of the CRU and specimen processing lab, and like many legacy environments, lacked automation, standardization, provenance capture, and efficiency, all of which are critical and essential for effective sharing.

### The C-BIG Solution

The Neuro developed a scalable Open Science model that facilitates collection, curation and sharing of multimodal data, the process of which could itself serve as a model for others. Existing workflows benefited greatly by adhering to the FAIR principles (Findable, Accessible, Interoperable and Reproducible) in their design (Wilkinson et al. 2016).

#### Organizational Factors

In order to build an infrastructure for institutional Open Science, a number of non-technical factors needed to be considered. Adoption of Open Science followed an eighteen-month stakeholder consultation, which resulted in a framework for the institute structured around five “pillars”. One of these pillars was focused on intellectual property. The Principal Investigator (PI) community at the Neuro agreed that the innovations of the institute were almost exclusively considered basic science, or foundational discoveries, and therefore agreed that the PIs at the institute would forgo institutional support to establish intellectual property. Another essential step was to ensure a proper governance model in which all key stakeholders agreed about the implementation details. This is one of the most difficult challenges, given the implementation permutations and, therefore, divergent views on which is best. Strong leadership with clear vision of the end product is key to unifying the various perspectives. We relied heavily on established best practices and existing software that specialized in data sharing, adapting the various workflows to the functionalities of the software.

Once aligned on an implementation roadmap, which was done with regular meetings and subcommittees working on specific problems, it was imperative to ensure that those researchers supplying C-BIG with data were also aligned with the overall vision and implementation details. We tackled this one issue at a time, beginning with sample tracking, moving to the handling of sample metadata, identifiers, followed by processing and analysis, and ultimately bridging this to other domains and modalities. Another key pillar in the implementation of our Open Science mission is the principle of autonomy, meaning that individual PIs had to opt-in to the Open model, and that the institute not forcibly mandate data sharing. As such, our model centers around creating tools and workflows that are efficient, robust and feature-rich so that researchers themselves see the value in sharing data. This is critical in ensuring continuous data flow into the system, and as such requires translation, education and training to ensure researchers understand exactly how to use the system and configure it to their workflows. We began this interactive and iterative process with the CRU, then proceeded with specific candidate projects, such as Parkinson’s and ALS, and are currently expanding to more use cases, including multiple sclerosis, neurocritical care, and neuro-oncology.

We were fortunate at The Neuro where institutional funding was available for this initiative, especially since Open Science in the life sciences is still relatively rare, with The Neuro being one of the first clinical-research institutes to undergo this transition. Despite the fact that a great deal of data sharing expertise resided in our group, implementation at this scale was still a challenge and required significant coordination and forethought.

#### Redefining Governance and Ethics

C-BIG established a novel governance framework reflecting innovative and forward-looking patient-centered ethical standards and best practices to protect patients and support ongoing patient participation in this initiative, while ensuring its utility as an Open Science resource. C-BIG’s high rate of recruitment of >98% demonstrates that patient enthusiasm has been encouraging, and shows strong patient and public support for Open Science.

At the institutional level, an Open Science ‘Leaders Council’ composed of a mixture of fortune 500 executives, scientists, academics, university officials, and community members, was formed. This committee meets on a bi-annual basis and reviews all of the institutional Open Science advances and progress on specific platforms (such as C-BIG). The local research ethics board (REB) required that a separate governance board, consisting of both internal and external members representing legal ethics, neuroinformatics, translational research, and patients, be struck to review the activities and practices of the C-BIG repository.

C-BIG data are made available as widely as possible to the scientific community for health-related research. The repository was designed to implement novel data access models along with privacy-preserving functionality and other ethics tools developed to protect the interests of individuals contributing data and biospecimens to Open Science (Dyke 2018 & 2016b). For example, the initiative expanded Consent Codes, a structured way of recording consent permissions for the reuse of data in Open Science compute environments, to include biobanking-specific codes for the reuse of biospecimens (manuscript submitted for publication). Along with access to comprehensive metadata, these new Consent Codes will enhance researchers’ ability to identify C-BIG resources suitable for their research plans. The C-BIG framework was generalized (C-BIG-specific details were removed), a template for which was posted on the MUHC’s ‘Centre for Applied Ethics’ website (https://tinyurl.com/yyfswpcj).

#### System Design

System design is critical with considerable time spent not only internalizing existing workflows, but understanding how a system upgrade could leverage best practices in data sharing without disrupting daily workflows too severely. Using open source software that could easily be extended and adapted without proprietary licensing issues is an important consideration. As such, a centralized design for the internal infrastructure was specially chosen in 2017, leveraging the LORIS data management system as the technical backbone for this initiative due to its extensive history in managing longitudinal, multimodal project data.

A key benefit to upgrading workflows with newer software solutions is automation, given that it becomes increasingly cumbersome to share data in a scalable fashion if too many manual interventions are required. Extending LORIS to automate the existing Standard Operating Procedures (SOPs) at The Neuro was the first implementation task. It was imperative to respect the existing procedures of biospecimen annotation and handling, while simultaneously allowing interoperability with other data types, such as phenotypic measures or imaging studies performed at the McConnell Brain Imaging Centre (BIC) (another key unit of The Neuro).

#### Configuring the Database

To cause minimal disruption to clinical staff and existing workflows, configuration of study parameters needed to encompass the already existing dataset. A crawler was implemented to parse the proprietary database and automatically import previously stored data to C-BIG including the existing Projects, Subprojects, Sites, Visits, and Identifier formats, in addition to configuring some display settings. Once the base parameters of the study were established, the consolidation and centralization efforts began where each datapoint, whether clinical, imaging, genetic or biospecimen, relied on the preconfigured variables to be stored in the new database.

#### Creation of the Biobank Module

The Biobank design was the result of discussions with CRU personnel to understand their processing requirements, current workflows, and opinions of existing systems. Consequently, the Biobank module includes an intermediate level Laboratory Information Management System (LIMS) designed for live laboratory data entry, equipped with a barcode scanner interface and the ability to be used offline, while also linking to other datasets across LORIS (Rabalais et al. 2019), with source code openly available at https://github.com/aces/biobank_wg. Specifications defined by the Standard PREanalytical code (SPREC) are adopted to enable effective interconnectivity and interoperability between any research project that conducts biomedical research (Betsou et al. 2018). Accordingly, the Biobank schema implements mandatory fields with strict data types that define specimens based on type, primary container type and storage conditions [Table 1]. This allows the module to manage and track factors impacting biospecimen integrity and is fundamental in providing research quality samples. Specimens are managed within the context of LORIS through association with patients, visits, projects and sites.

**Table 1:** Example of metadata fields shared across all specimens in the database

Field	Value
*Type*	Serum
*Container Type*	Cryotube Vial
*Patient*	TOSI0000001
*Visit*	Visit 01
*Projects*	TOSI
*Current Site*	CRU-MNI
*Quantity*	500 μL
*Freeze/Thaw Cycle*	0
*Temperature*	−80°
*Status*	Available

A key Biobank feature is to record different data for specimens based on their processing requirements. Correspondingly, a portion of specimen metadata for processing is recorded using variable-key attributes that are associated with specific SOPs [Table 2]. Values are then linked to those keys and stored using JavaScript Object Notation (JSON). This feature allows fields to be customized across specimen types and processing stages within a given project.

**Table 2:** Example of how key to SOP relations can differ across SOPs

Isolation of Serum from Whole Blood - SOP BB-P-0003		DNA Extraction from Whole Blood - SOP BB-P-0009	
key	value	key	value
Milky Serum	FALSE	DNA Quantification Date	2019-10-29
Hemolyzed	TRUE	DNA Concentration (ng/μL)	178.9
Hemodialysis Index	2	260/280 Ratio	1.86

The Biobank infrastructure is incorporated into the core LORIS database and allows data to be reliably joined via MySQL entities. This structure can then be interfaced with the Data Querying Tool for easy data extraction (MacFarlane et al. 2014). The Biobank utilizes the React Javascript Library^1^ for all operations performed in the web interface, which supports user interfaces that host complex and dynamic data with reduced response lag (Gackenheimer 2015).

Respecting FAIR principles, the Biobank relies on a RESTful API^2^ to query, submit, validate, and store data collected in the lab. This API-centric design makes it possible for any authorized user or project to access the data, and add or update data based on granular permissions. While users can perform any operation from the web interface, the structural implementation of APIs allows projects to transfer data to C-BIG more easily and with greater integrity conservation.

#### Consolidating Workflows

Designing a scalable system for institutional Open Science is more than a theoretical prototype. In practice, it involves consolidating the existing workflows with data sharing best practices in a manner that is not too disruptive, but is also transformative. For C-BIG, this was an interactive process that involved ongoing and continuous discussions with numerous working groups in order to integrate key subcomponents, including the 1) consenting process, 2) biospecimen workflows, 3) clinical workflows, as well as incorporating genetic and neuroimaging data.

**Fig. 2:**
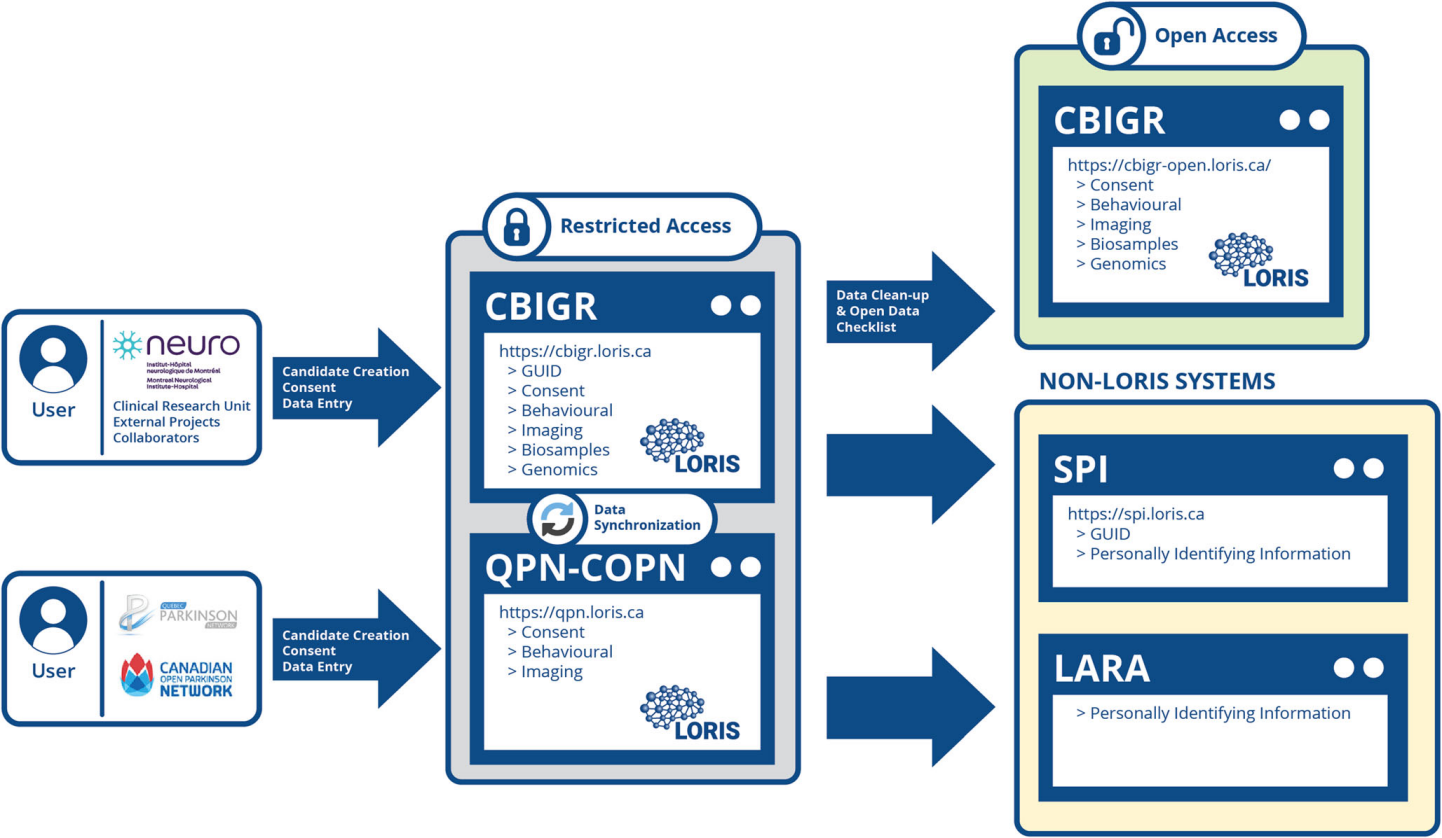
Data flow and interoperability chart between the various subsystems

1. Consenting process: Beginning with recruitment, CRU personnel are instructed to collect a patient’s personally identifying information (PII) and consent before performing clinical, neurocognitive tests or acquiring biospecimens. A recruitment tool was implemented to enable CRU staff to enter all collected information in a single location. While the patient’s PII can in no circumstances exist on the same server as the patient’s data collected for wider research analysis, it is still critical to store that patient information within a clinical environment to keep consent information current while continuing to collect new data. To that effect, a parallel application called the Sensitive Patient Information (SPI) system was developed with the sole purpose of storing PII in a secure location and linking it, through an untraceable patient ID, to the clinical and biosample data stored in C-BIG. This procedure prevents data entry errors during recruitment and streamlines the work of the CRU staff, while abiding by the ethics regulation to keep patient information completely segregated from their clinical data, both virtually via firewalls and physically in the server room. In order to submit the data separately, but simultaneously, to the SPI system and the C-BIG database using a single entry form, an elaborate algorithm generates a unique untraceable patient identifier (Das et al. 2017) upon form submission. The algorithm then parses the form to selectively submit each field to their respective databases, leaving the identifier as the sole variable that the two systems have in common, thus dramatically reducing the risk of compromising a patient’s identity.
2. Biospecimen workflows: When C-BIG receives patient samples collected from, and de-identified by, the CRU, they are then processed by lab technicians as per C-BIG SOPs and stored in the database (Fig. 2). C-BIG has CTRNet^3^ certification and all personnel are educated and trained on national biobanking standards. C-BIG receives samples in the form of whole blood, saliva, cerebrospinal fluid (CSF) and biopsies, which are then processed into a variety of derivatives including peripheral blood mononuclear cells (PBMC), serum, plasma, DNA, RNA, fibroblasts, muscle tissue and inducible pluripotent stem cells (iPSC). Once processed, uniquely identifiable barcodes are assigned to the specimen vials, which are frozen in long term, ultra low temperature freezers or liquid nitrogen tanks. The biobank system is then used to track their inventory location and key metadata associated with each of the samples.

3) Clinical workflows: Clinical data collection was less affected by the system upgrade. Patients are still required to undergo an onsite visit where data is collected on paper forms and subsequently digitized on custom designed LORIS instruments mimicking the paper format. This limits errors with data collection, such as scores that fall outside the scientifically accepted scales in answers collected by the paper forms and the webform. During their visit, patients also provide informed consent to their participation in C-BIG so that their data and biospecimens can be analyzed and distributed to researchers.

#### Genetics

Genetic data was independently processed and genetic variants results incorporated back into the database. The initial goal was for C-BIG to ingest and export annotated gene variants, while making the data queryable alongside clinical, imaging and biospecimen data. Several solutions were required to make this possible. The first challenge was to transfer gene variants files (vcf files) to the database server to read and parse the file. We solved this issue by leveraging the Neurohub infrastructure (https://neurohub.ca) to synchronize files between multiple computers. Once a file was accessible by the C-BIG server, an algorithm was devised to deconstruct it into several smaller meaningful subsections to be partially reconstructed. This reconstruction occurs when researchers query the genetic data in combination with other modalities and apply filters on their results. To offer greater querying flexibility, resulting in a file solely containing user specified patients, the system is capable of recombining the variants data for any set of patients independently from filtered out patients. Once satisfactory results are obtained, and if the results contain genetic data, the results can be exported back using the Neurohub architecture and synchronized across other computers as it was done for the original file transfer.

#### Neuroimaging

Neuroimaging data are also a key component of The Neuro’s Open Science mission. For C-BIG, data can be uploaded directly from BIC scanners using existing functionality for preprocessing and validation. Once uploaded and inserted in the database, data is directly linked to other existing data types for any given patient, and viewable directly. C-BIG will take advantage of new LORIS functionality for electrophysiological signal data for EEG and MEG. Since 2015, LORIS has supported MEG data (Niso et al., 2015) and integrated EEG-BIDS (Madjar et al., 2018; Pernet et al. 2019; Bosch-Bayard et al. 2020) as well as iEEG-BIDS (Frauscher et al., 2018) data. MEG-BIDS (Niso et al., 2018) is currently being implemented. Cross-linking these temporally precise modalities with anatomical and functional images will enrich C-BIG analysis options.

#### Robust Testing

As part of quality assurance, C-BIG was subjected to rigorous testing for deployment [Table 3].

**Table 3:** List of tests that were conducted to ensure proper functionality of the C-BIG system

Test type	Details
System configurations	Routinely run tests based on validated database configurations and imported study parameters.
Atomic operations	Every atomic Biobank operation^1^ was tested independently at each test deployment.
Biobank integration	Operation combinations^2^ were tested with acceptable samples of permutations succeeding.
Biobank usability	Frequent usability tests were always performed by clinical users to ensure intuitive development.
Scalability testing	The system was loaded with high flux of data to identify and correct algorithmic inefficiencies.
Integration testing	The entire system was tested using real scenarios. Deployment contingent on a 100% passing grade.

^1^Atomic operations are unique actions users can take in the module (add a new biospecimen, edit a container, discard used pool)

^2^Operation combinations refer to a sequence of ordered atomic operations (create sample, pool it, aliquot the pool and discard it)

Once all tests passed, C-BIG entered BETA testing, during which CRU personnel and lab technicians were instructed to mirror all operations on prior systems to become trained on the new system and identify any remaining flaws. The system completed BETA testing in November 2019 and was launched internally the same month.

Since the launch, the latest security patches and LORIS updates have been applied to the system. These improvements are generally published as soon as a flaw is discovered, and do not require testing as they are very targeted towards specific code sections. Major LORIS releases are also applied every few months to improve the user experience, offer new features, and optimize the database, where similar testing outlined in Table 3 is applied.

#### Documentation

For any successful software initiative, proper documentation is imperative, reducing the learning curve and increasing community adoption. A manual of operations was created for C-BIG (https://tinyurl.com/y63ry87g). C-BIG will also link to other key documentation created from the various subcommittees, with responsive help sections available throughout the portal.

#### Data Mapping

Once the various design choices were made, with workflows fully internalized, and a unified software environment configured and deployed, the task of carefully mapping data was tackled. For C-BIG, a plan was thoroughly discussed to ensure a clean execution of the transfer and consolidation of data from the legacy systems. This required a number of specific operations that were dependent on the nature of the data being transferred, and are outlined below:

A) *Patients & Visits:* Each legacy system had its own patient lists that needed to be consolidated and harmonized. Data crawlers were created to extract patients and visit information with imported data compared to remaining legacy systems for discrepancies.
B) *Clinical data:* Once data was imported to the new system, the digitization of the clinical data began. CRU personnel were required to manually enter the content of the paper forms into the LORIS instruments. Digital data entry allowed users to track the progress of patients, and enabled searching for specific patients based on specific clinical criteria.
C) *Biosamples:* Given the lack of integrity constraints in the proprietary database, new automation scripts validated specimens and autocorrected samples that failed (bad formatting, incoherent sample types, missing/unnecessary specimen attributes, etc.).

#### The Public Layer

Setting hard boundaries between access levels was the key concern and challenge. Open access data is a subset of the registered data, which in turn is a subset of the restricted data available to the internal C-BIG staff. In order to have complete control over the data at each tier, an exhaustive list of variables was generated from LORIS with each variable individually assigned an access level: open, registered or restricted. The architecture supporting this tiered access is composed of three separate databases. The LORIS interface opens a connection to the appropriate database after validating the user’s role, whether an internal staff member, a registered researcher, or an individual simply accessing the open portal. Data in these three tiered databases is imported directly from the main LORIS data source, and is never altered within the different tiers after it’s import. Instead, a regular synchronization procedure ensures that the databases are updated with any amendments applied on all of them from the source.

## Results

The C-BIG repository is a combination of a number of components: a digital biobank for data archiving and sharing, a physical biospecimen repository, a multi-level access web portal, a patient registry, a de-identification layer, and an API for automated interactions. Together these feature an ecosystem that links and shares multimodal data from numerous labs and projects, spanning several neurological diseases. This design concept will aggregate multimodal neuroscience datasets of tremendous scientific value which are available to the research community. The first public data release that occurred in November 2020 included multi-modal data from several patient cohorts including Parkinson’s disease, ALS, and neuromuscular disease. This ecosystem will continue to add and disseminate a growing number of patient biospecimens and associated patient-data as they are collected. This collection is expanding to include pathologies such as multiple sclerosis, frontotemporal dementia, and autism / intellectual disability. Thus far, three key results have been achieved: i) a software infrastructure that has been built specifically to disseminate and share data in an automated and structured manner, ii) a significant and growing collection of biospecimens and associated multimodal data, and iii) a model for other institutions, who are interested in following suit, to operationalize.

### The C-BIG Portal and its Functionality

Effective sharing begins with efficient and structured data acquisition. The Biobank serves as a data entry point and digital registry for patients who provide informed consent in the CRU. It is specifically tailored to the specifications of the physical infrastructure and processing workflows, streamlining the data acquisition process in an accessible, queryable and shareable manner.

#### The Internal Biobank Infrastructure

**Fig. 3:**
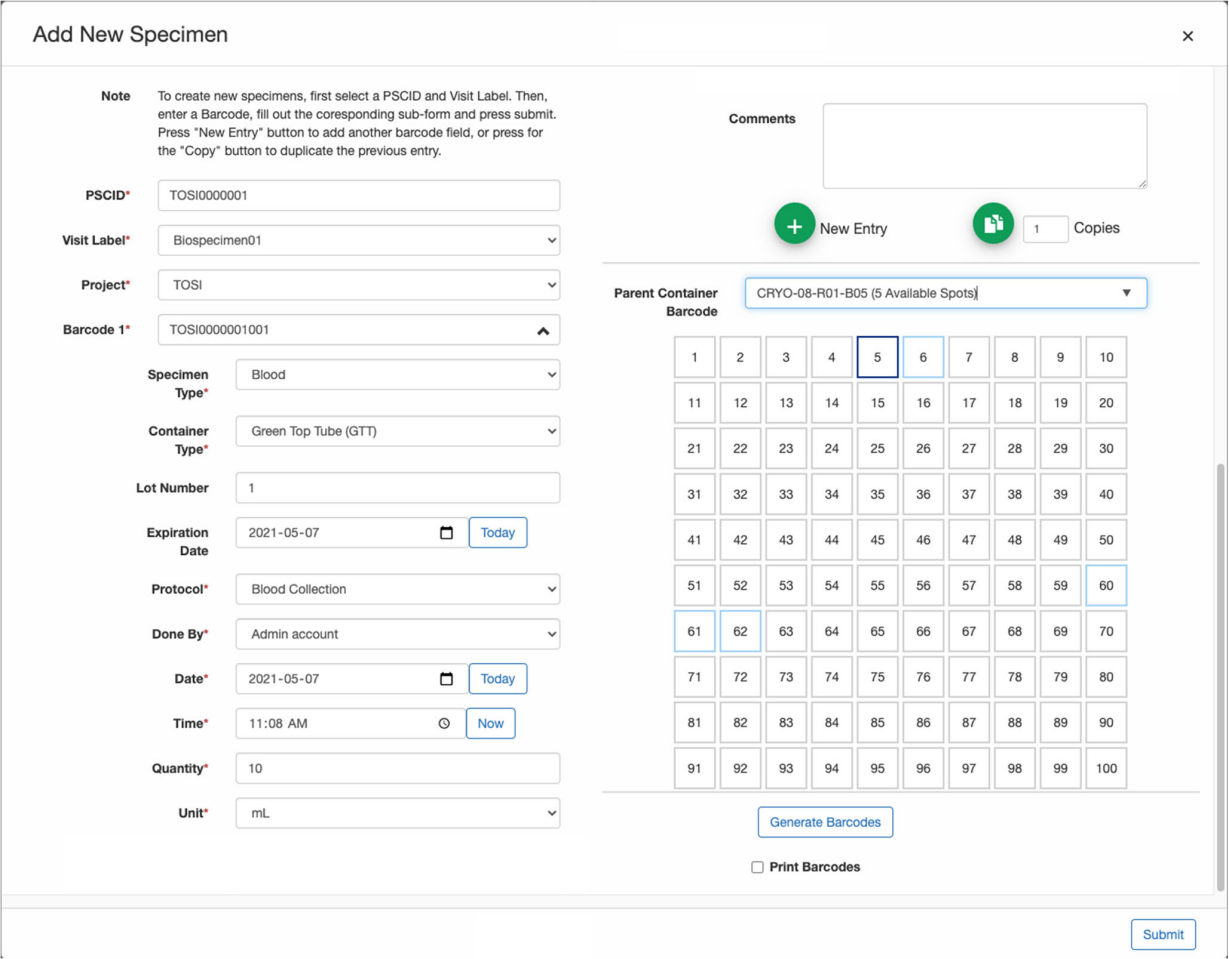
Biobank data entry form for biospecimens

The internal Biobank infrastructure facilitates acquisition relating to both stages of biospecimen processing: collection and preparation. The entry point for specimens is a form that records information needed to define and associate them to a patient, visit, project, container type and specimen type (Fig. 3), all of which can be batch imported as well. The chosen specimen type determines the available SOPs, which when selected, dynamically generates fields specific to the SOP. A unique barcode is issued and printed for each created specimen. The label is then attached to the specimen’s primary container and used throughout the system to track, query or share the specimen data. The form also provides the opportunity to directly assign the newly created specimens to a matrix box container for storage.

Once collected and registered in the system, specimen data is presented to the user in a table that can be filtered to quickly access specific subsets of specimens according to recorded attributes, and downloaded for further analysis. Specimens can also be aliquoted into derivative samples, pooled together into a single sample, batch prepared or even edited concurrently with specimens that share similar properties.

**Fig. 4:**
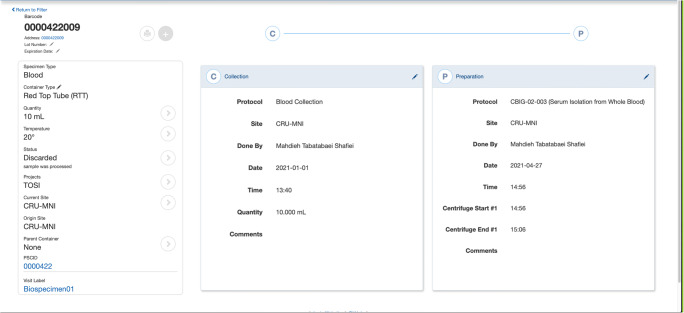
Biobank specimen page displays specimen metadata, processing stages & life cycle

An individual specimen’s data and metadata can be viewed, added or manipulated through a variety of forms, fields, and interfaces (Fig. 4). The left side of the page displays the global parameters that define the specimen, while the right side displays all the attributes related to processing stages of a specimen’s life cycle.

**Fig. 5:**
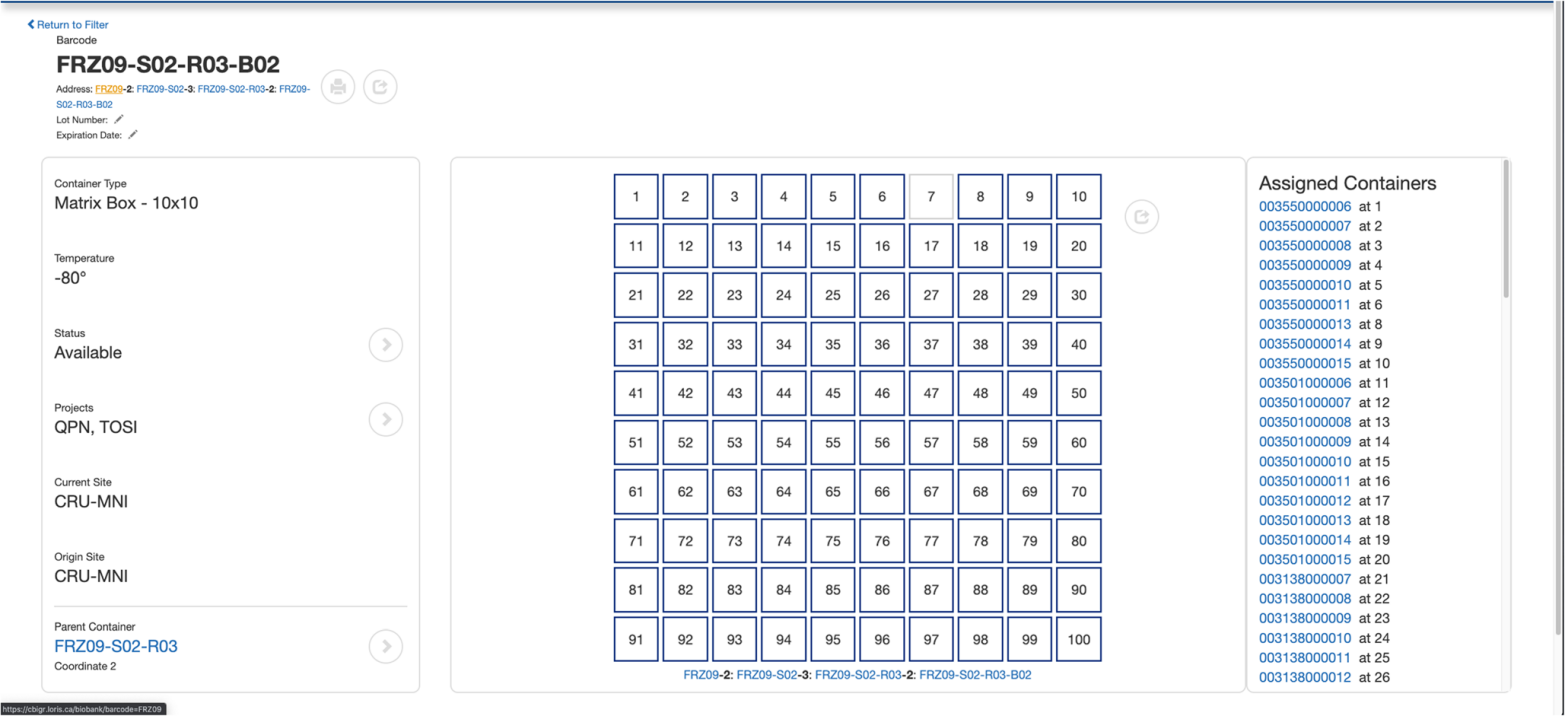
Biobank container page with graphic display of the container dimensions and contents

Specimens are stored in hierarchically nested containers with configurable dimensions, each displayed on their own page with global attributes on the left, a list of sub-containers on the right, and a graphic visualization in the center that facilitates location assignment and access (Fig. 5). Location is assigned by dragging and dropping specimens into the desired container, by changing the container’s parent, or by loading subcontainers sequentially using a barcode scanner. Navigating down the container hierarchy requires clicking on centre boxes, or on any of the barcodes listed on the right. Navigating up the hierarchy is accomplished by clicking on the parent container barcode. These functionalities allow both containers and specimens to be quickly accessed, stored, and moved within the Biobank.

#### Validation

Precautions are built into the system to ensure the integrity of the data entering the digital repository, such that every field is required to pass a multilayered validation pipeline to reduce risk of human error. The first layer of validation relies on the conditional display of fields based on the selected SOP described in section 3.1.1 above. This, in combination with the LORIS permissions system, prevents the user from entering specimen data incoherent with the specimen type, the processing protocol or the patient’s site and project affiliations. The second layer is client side Javascript and HTML code, where data type errors are flagged, and lists and ranges are validated. The third layer involves cross-matching entries with the database values across all other samples, as well as redundantly validating data-types to prevent malicious data corruption attempts. Upon submission, invalid specimens are flagged and rejected with a descriptive error message provided to the user and therefore never enter the database. Conversely, valid specimens that enter the database are tracked via audit logs that are regularly backed up. Finally, since automated validation can not always prevent human entry errors, it is standard procedure for the C-BIG laboratory team to regularly review entries in the system and cross check them against the collection and processing records from the lab. It should be noted that a granular permission scheme restricts a user’s breadth of data access, as well as their permitted operations, based on several factors such as their role, site and project affiliations.

#### The C-BIG Public Interface

Designing an Open Science system like C-BIG required careful thought about interoperability at multiple levels: i) between internal workflows, ii) with external units, iii) using existing software and hardware, iv) amongst researchers and their analysis habits, or v) with other data sharing platforms. The public layer, launched in November 2020, is a manifestation of the various elements and workflows that have been developed to disseminate data to the larger research community.

Data, metadata, and summary statistics are available to users in a number of ways. First, comprehensive analytics outlining the full range of modalities, disease types, and participating studies are openly and easily available on the Dashboard, and can serve as a first step towards exploring the data. To perform more refined searches, a web based querying engine enables researchers to filter multimodal data across studies, sub-cohorts, or any other field of interest, and contains a complete data dictionary for every queryable parameter. Searching through the data can therefore be extremely granular, with the data organizable, and re-organizable, as per any variable in the database. To facilitate analysis, the queried data can be collated longitudinally across the lifetime of data acquisition, or cross-sectionally over each variable. Queries can be saved conveniently for future use, with queried data downloadable into a number of formats directly from a web browser. The querying tool also supports batch downloading of imaging datasets that are also viewable in high resolution through the portal. Lastly, a desired objective for the querying engine is to expose annotation tools, currently being used by technical staff to curate the data, and to eventually crowdsource quality control.

Due to privacy risks and restrictions on permitted uses of data, C-BIG will have several tiers of access making it possible for bona fide researchers to access restricted data and request associated biospecimens. Complete documentation on the composition and accessibility of these datasets will be published via the C-BIG portal (https://cbigr-open.loris.ca) with detailed instructions (https://tinyurl.com/y63ry87g) for use and download. Data and any various associated metadata will also be available via the Canadian Open Neuroscience Platform (CONP) portal at www.conp.ca (Accessed 24 August 2020).

### The Data

**Fig. 6:**
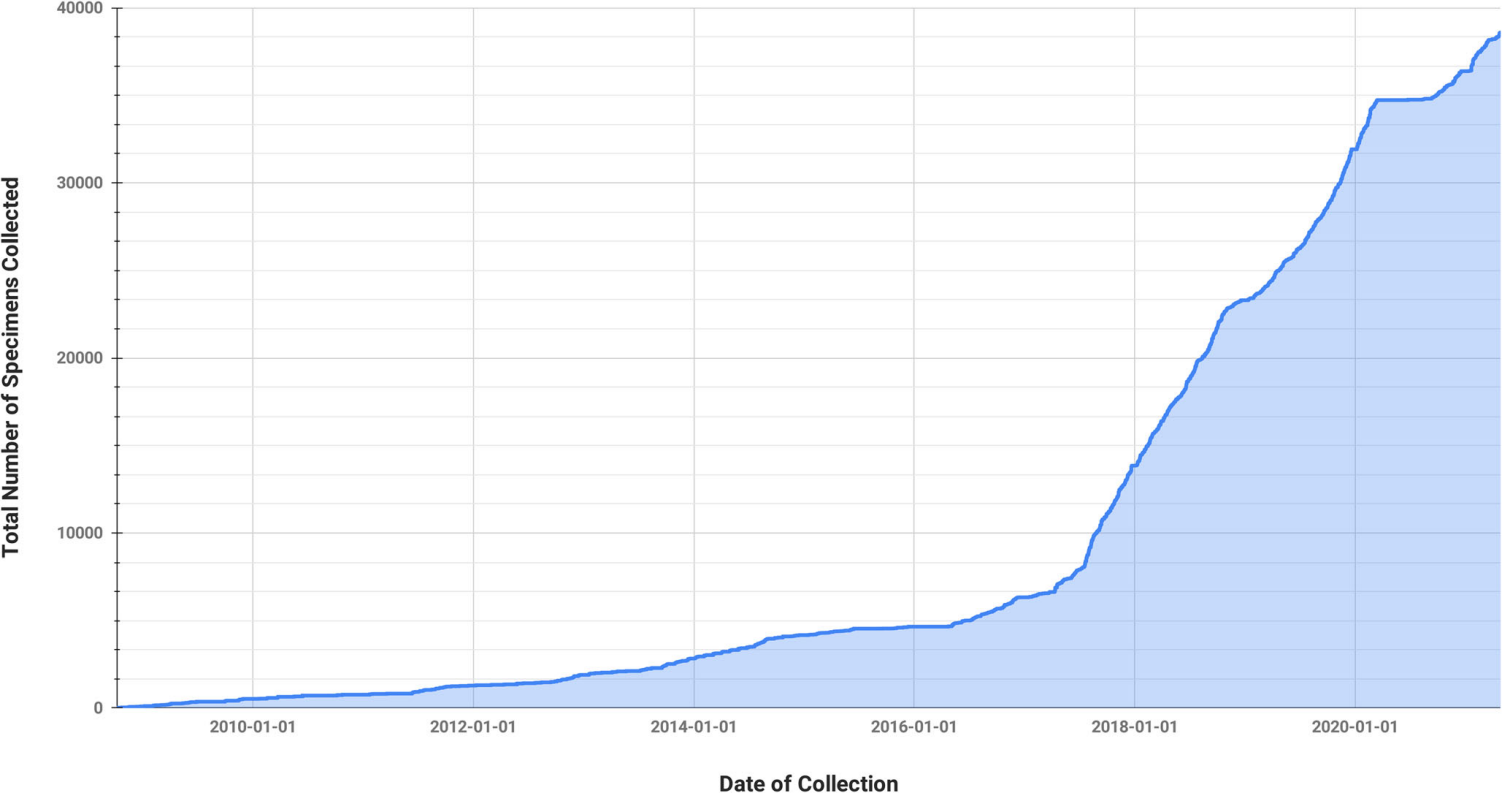
The number of biospecimens collected over time in the C-BIG since 2008

C-BIG currently houses over 32,500 available biospecimens from more than 80 disease cohorts (Fig. 6), with ongoing data and specimen acquisition from patients and healthy controls. All of these samples have been carefully organized within a unified database and are accessible to researchers at The Neuro. Beginning in November 2020, data is gradually being made accessible to the greater research community in the form of analytics, metadata, as well as specific datasets. The first data release was an institutional decision to target specific disease populations and will begin with Parkinson’s Disease (PD), Amyotrophic Lateral Sclerosis (ALS) and Multiple Sclerosis (MS), with plans to scale with other disease populations.

**Fig. 7:**
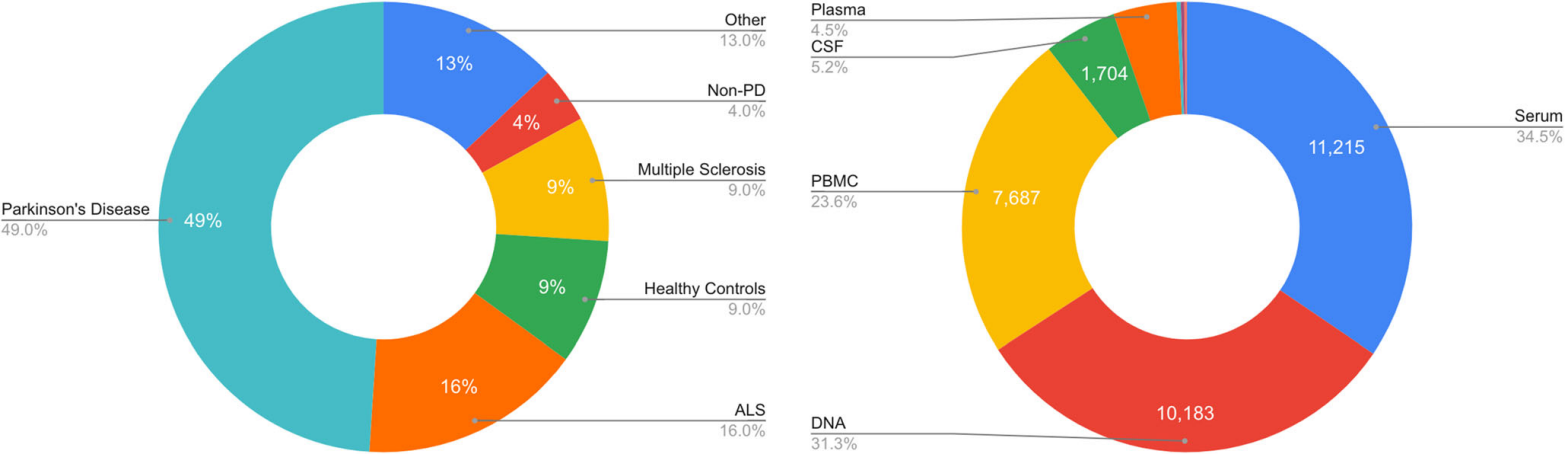
(LEFT) C-BIG currently houses data from 1720 patients (931 males/789 females) in 88 disease groups across 20 projects and 19 sites, 49% with a clinical diagnosis of Parkinson’s disease. (RIGHT) Over 32,500 biospecimen samples have been collected and archived in C-BIG. The storage infrastructure includes 367 Matrix Boxes, and 12 Freezers and Cryogenic Tanks

Each patient registered in C-BIG is associated with a disease cohort (Fig. 7 LEFT). Parkinson’s Disease is by far the largest of the groups, accounting for 49% of all patients. Cohorts with Amyotrophic Lateral Sclerosis (ALS) and Multiple Sclerosis (MS) also make up a significant portion of the diagnosed patients, accounting for 16% and 9% respectively. Patients with Parkinson’s Disease have contributed over 18,000 of the available samples, while ALS patients have donated nearly 9000 samples and MS patients have provided over 4000. Of the available specimens in C-BIG, Serum (34.5%), DNA (31.3%) and PBMC (23.6%) account for the majority (Fig. 7 RIGHT). The repository also stores CSF, Plasma, Fibroblasts and iPSC samples. The total number of iPSCs is currently 97, comprising 20 healthy control lines, 13 PD patients, 20 ALS patients, 4 ID/ASD patients, 20 CRISPR gene KO, and 20 CRISPR edited. The CRISPR KO are lines in which disease-relevant genes have been knocked out, while the CRISPR edited are lines in which a mutation was corrected, or where the mutation was added.

C-BIG currently houses several key metadata parameters from 585 sequenced patients from the Canadian Open Parkinson’s Network (https://copn-rpco.ca) [Table 4]. Additional parameters are being made available on request.

**Table 4:** Metadata for 585 sequenced patients in 8 different disease groups

Disease Name	Amount of Patients Sequenced
Atypical Parkinsonism	4
Essential Tremors	1
Gaucher Disease	1
Lewy Body Dementia	3
Multiple System Atrophy	2
Parkinson Disease	568
Progressive supranuclear palsy	5
Wilson Disease	1

Imaging data is also a key modality of this repository, comprising an initial cohort of 81 MRI and 94 MEG scans from the Quebec Parkinson’s Network (http://rpq-qpn.ca) (Gan-Or et al. 2020). Data from the BIC will flow directly into C-BIG in an automated manner and will significantly increase the number of scans in the database, including other modalities such as EEG.

### An Institutional Model for Sharing in a Clinical Research Setting

The process for releasing data was not a simple transition, but rather years of careful preparation and design. Not only did this process span multiple population cohorts, it also involved different committees and working groups, and required upgrading existing workflows with new technologies. A key deliverable from this significant undertaking is a stepwise log for other institutions looking to similarly transition. A complete guide can be found at https://loris.ca/MCINOpenScienceGuidance_DataPrepChecklist.pdf. While not every step may apply equally to every institution, the general components will surely be similar elsewhere. The Neuro has committed to this philosophy with the thought that the more data is shared with a greater number of collaborators all over the globe, scientific discovery will be accelerated.

## Discussion

An increasingly evident impediment to modern science is the inability to share data or reproduce experimental results (Open Science Collaboration 2015; Pashler and Wagenmakers, 2012; Fanelli 2018, Baker, 2016). Making data available with as few barriers as possible is largely becoming a requirement, and as such, many funding agencies are beginning to mandate data sharing plans, especially as they consider the cost of data loss with legacy procedures (Poldrack & Gorgolewski 2014). Making data openly available has not only become easier in recent years, but also has allowed for larger groups and consortia of researchers to perform analysis. Furthermore, as machine and deep learning techniques are beginning to mature, the need for larger sample sizes of easily accessible data is becoming stronger (Button et al. 2013; Turner et al. 2018). Filling out a multitude of bureaucratic data access agreements is not a viable option when using these valuable analysis methods, and ultimately impedes collaborations.

It is important to note that while simply “going open” is an important step, there are numerous considerations to take into account, including proper resourcing and a realistic transition plan. The reality of the transition plan at The Neuro was convoluted and required strong oversight. It involved numerous committees, working groups, meetings, design iterations, and discussions. Transitioning from traditional science protocols to taking advantage of web technologies and best data sharing practices was a tricky task, especially given The Neuro’s pioneering role in the community. A great deal of work remains in terms of scaling, interoperability and standardization to fully facilitate the transition to Open Science, however beginning the process is key to making more datasets available to the greater research community.

Currently, there is an increase in open datasets released to the community, but institutional adoption is still lacking. Some institutes like the Allen Institute (https://alleninstitute.org) have openly released datasets, such as notable templates and atlases, whereas others have created frameworks dedicated to open sharing, such as OpenNeuro (https://openneuro.org), Canadian Open Neuroscience Platform (https://conp.ca), and Data Commons (https://datacommons.org). There are also environments that facilitate open sharing, such as Open Science Framework (https://osf.io), Zenodo (http://zenodo.org), and DataLad (https://datalad.org). Most of these initiatives provide specific functionalities to the scientific community, and are themselves evolving with research best practices. Regardless of what solution is adopted to facilitate scientific data sharing, one key consideration for all platforms is interoperability and standardization in order to fulfill the larger goal of globalized data sharing. As such, we have engaged with numerous data sharing initiatives, best practices committees, and standardization groups to include the latest improvements. C-BIG offers a unique approach by releasing clinical research data built around biospecimen and patient data that continues to grow, both in terms of consented patients, and with regard to longitudinal patient followup.

### Future Directions

Following the initial launch of public data in November 2020, further development to the system has been planned to take place. These include the following, listed below.

1. *Data querying optimization:* Scalable querying is being developed based on query sizes, data types, and result sizes, and then choosing the most efficient dissemination method.
2. *Better user experience (UX):* Frequent software updates will result in faster navigation, a more intuitive interface, shorter waiting times and a smoother learning curve.
3. *Greater standardization*: ReproNim form generation (Kennedy et al. 2019), and Reproschema standardization (Abraham et al. 2019) will improve our Instrument Builder.
4. *Genomic expansion:* Enhanced capabilities to parse, analyse, and extract meaningful information from gene sequencing files is currently underway.
5. *High Performance Computers*: Cyclical workflows (data transfer, processing and analysis) of genetic/imaging data is being enhanced (Sherif et al. 2014; Das et al. 2018).
6. *Advanced analytics:* Summary statistics for modalities, total numbers, key measures will be enhanced, as well as features to facilitate machine and deep learning.
7. *Biobank optimizations:* Improvements with data streaming, CPUs, React state management, database requests, and regular user feedback for intuitive usage.
8. *Biobank Shipping Module:* Capturing the full chain of custody of a sample during transit will facilitate communication and maintain overall data integrity.
9. *Data querying API:* RESTful interface to for complex query building allowing for direct scripting of queries without depending on the web interface.

## Conclusion

Aggregating biological materials and high quality multimodal patient data via accessible technology platforms is essential to advancing scientific understanding of neurological and human diseases. With C-BIG, we are making it easier to do clinical and translational research, accelerating the ability to collect and share valuable datasets and patient biospecimens with the goal of improving patient care. The C-BIG repository will continue to evolve with new studies and growing datasets, providing greater statistical power for investigations into the biological mechanisms driving neurological diseases. Supported by an internationally recognized research institution, and with interoperability and standardization as its core design principles, the platform will continue to scale across data types and analysis techniques, with the hopes of paving the way for other institutions to follow.

## Footnotes

1 https://reactjs.org/

2 https://www.ics.uci.edu/~fielding/pubs/dissertation/rest_arch_style.htm

3 https://www.ctrnet.ca/
